# Activated Microglia Inhibit Axonal Growth through RGMa

**DOI:** 10.1371/journal.pone.0025234

**Published:** 2011-09-21

**Authors:** Mari Kitayama, Masaki Ueno, Toru Itakura, Toshihide Yamashita

**Affiliations:** 1 Department of Molecular Neuroscience, Graduate School of Medicine, Osaka University, Osaka, Japan; 2 Department of Neurological Surgery, Wakayama Medical University, Wakayama, Japan; 3 Graduate School of Frontier Biosciences, Osaka University, Osaka, Japan; 4 Core Research for Evolutional Science and Technology (CREST), Japan Science and Technology Agency (JST), Tokyo Japan; Hokkaido University, Japan

## Abstract

By causing damage to neural networks, spinal cord injuries (SCI) often result in severe motor and sensory dysfunction. Functional recovery requires axonal regrowth and regeneration of neural network, processes that are quite limited in the adult central nervous system (CNS). Previous work has shown that SCI lesions contain an accumulation of activated microglia, which can have multiple pathophysiological influences. Here, we show that activated microglia inhibit axonal growth via repulsive guidance molecule a (RGMa). We found that microglia activated by lipopolysaccharide (LPS) inhibited neurite outgrowth and induced growth cone collapse of cortical neurons in vitro—a pattern that was only observed when there was direct contact between microglia and neurons. After microglia were activated by LPS, they increased expression of RGMa; however, treatment with RGMa-neutralizing antibodies or transfection of RGMa siRNA attenuated the inhibitory effects of microglia on axonal outgrowth. Furthermore, minocycline, an inhibitor of microglial activation, attenuated the effects of microglia and RGMa expression. Finally, we examined whether these in vitro patterns could also be observed in vivo. Indeed, in a mouse SCI model, minocycline treatment reduced the accumulation of microglia and decreased RGMa expression after SCI, leading to reduced dieback in injured corticospinal tracts. These results suggest that activated microglia play a major role in inhibiting axon regeneration via RGMa in the injured CNS.

## Introduction

Spinal cord injuries (SCI) often have devastating impacts on neural function, leading to reductions in motor and sensory abilities. These can be compensated for via regeneration of neurons and their axons; however, axonal regeneration in the adult central nervous system (CNS) is quite limited due to the presence of a number of axon growth inhibitors. These include myelin-associated proteins expressed by oligodendrocytes and chondroitin sulfate proteoglicans expressed by astrocytes [1]. Over the past decade, a number of studies have examined whether inhibition of these glial factors is a viable option for treating CNS injuries. Although these methods did enhance functional recovery to some extent [2], [3], the treatments were by no means uniformly successful.

SCI causes extensive inflammation and the invasion of a large number of microglia/macrophages to the epicenter of the lesion. It is currently unclear whether this influx of cells plays a protective or a detrimental role during recovery [4]–[9]. In support of the latter possibility, recent evidence has indicated that, along with myelin and glial scarring, activated microglia/macrophages are one of the major inhibitors of axonal regeneration. For example, activated macrophages have been shown to induce retraction of dystrophic axons, both in vitro and in vivo [10]. It was further demonstrated that MMP-9 inhibitor and chondroitinase ABC prevented macrophage-induced axonal retraction [11]. Additionally, dieback of injured axons was suppressed following treatment with minocycline, which inhibits activation of microglia/macrophages [12]. However, the key molecules involved in these processes have yet to be determined.

One group of candidates is the repulsive axon guidance molecules, which play an important role in precisely directing the navigation of growing axons during neural development. These molecules are expressed or re-expressed after adult CNS injuries and inhibit regeneration of the injured axons [13], [14]. In addition to astrocytes and oligodendrocytes, microglia and macrophages express guidance molecules that retract the axons, including Slit, Netrin-1, and repulsive guidance molecule a (RGMa), in the injured spinal cord [15], [16]. Of these, RGMa is particularly interesting. It is a glycosylphosphatidylinositol (GPI)-anchored glycoprotein that was originally identified as the molecule that collapses the growth cone and repels axons during development [17], [18]. RGMa expression increases after SCI, during which time inhibition of RGMa enhances axonal growth and motor function recovery [16].

In this study, we aimed to identify the role of microglia in axonal regeneration and its underlying molecular mechanism. We found that microglia mediate the inhibition of axon growth, and that this process involves RGMa.

## Materials and Methods

### Cell culture

Neurons were harvested from the cerebral cortices of C57BL/6J mice (Charles River, Yokohama, Japan) at embryonic day 18 (E18). Cortical cells were dissociated by incubation with 0.25% trypsin and 0.5 mg/ml DNase (Sigma-Aldrich, St. Louis, MO) for 15 min at 37°C, after which they were washed and triturated in DMEM containing 10% fetal bovine serum (FBS). The neurons were cultured with DMEM supplemented with 10% FBS and 1% penicillin/streptomycin in poly-l-lysine-coated dishes at a density 1×10^5^ cells/ml.

Primary microglial cells were obtained from C57BL/6J mice on postnatal day 3 (P3) as previously described [19]. Briefly, the cerebral cortex of each mouse was digested with 0.25% trypsin and 0.5 mg/ml DNase for 15 min at 37°C. Cells were passed through a 70-µm nylon mesh. The resultant cell suspension was diluted with 10% FBS/1% penicillin and streptomycin/DMEM and seeded into poly-l-lysine-coated dishes. After 10 days, the dishes were shaken so that floating microglial cells could be collected from the astrocyte-monolayer sheet and then cultured in 10% FBS/1% penicillin and streptomycin/DMEM at a density 1×10^5^ cells/ml. In this assay, more than 95% of the cells were CD11b-positive microglial cells.

Bone marrow-derived macrophages (BMDM) were obtained from bilateral femurs of adult C57BL/6J mice as previously reported [20]. Marrow cores were flushed using syringes filled with RPMI1640/10% FBS. After trituration, cells were washed once in media, then plated and cultured in RPMI1640 with 10% FBS, 1% penicillin/streptomycin, and M-CSF (50 ng/ml; Sigma-Aldrich). Non-adherent cells were collected at day 4. In this assay, more than 95% of the cells were CD11b-positive macrophages.

### Neurite outgrowth assay

Cortical neurons were co-cultured with primary microglia or BMDM on chamber slides (Lab Tek II; Nunc, Rochester, NY) or in transwell plates (pore size: 3 µm; Corning, Glendale, AZ) with 10% FBS/1% penicillin and streptomycin/DMEM. To activate primary microglia and BMDM, lipopolysaccharide (LPS; Sigma-Aldrich) was added to the culture at a concentration of 1 µg/ml. Rabbit anti-RGMa antibody (10 µg/ml; IBL, Fujioka, Japan; [16]) was added for the neutralizing assay. Microglia were treated with 20 µM minocycline (Sigma-Aldrich) to inhibit activation. The cells were fixed in 4% paraformaldehyde (PFA) and immunostained with a mouse monoclonal antibody recognizing βIII tubulin (TuJ1; 1∶1,000; Covance; Princeton, NJ) as the primary antibody and Alexa 488 anti-mouse IgG antibody (1∶500; Invitrogen, Carlsbad, CA) as the secondary antibody. The length of the longest neurite of each TuJ1-positive neuron was then determined using ImageJ software (National Institutes of Health, Bethesda, MD). Data were collected from 3 independent experiments, in each of which 100 neurons were counted per group. The length in each group relative to that of control (neurons-only) was then calculated.

### Collapse assay

To visualize growth cones, neurons were fixed for 20 min with 4% PFA and stained with rhodamine-conjugated phalloidin (1∶500; Invitrogen) and anti-TuJ1 (1∶1,000; Covance). Growth cones were designated as “collapsed” if they had lost lamellipodia and ≤2 filopodia [21]. Data were collected from 3 independent experiments, in each of which 50 growth cones were counted per group. To calculate the percentage of collapse, the number of collapsed growth cones was divided by the total number of growth cones.

### Lipofection with siRNA

Transfection experiments with mouse RGMa siRNA (stealth siRNA, Invitrogen) and negative control siRNA (Applied Biosystems, Tokyo, Japan) for primary microglia were performed using Lipofectamine 2000 (Invitrogen) according to the manufacturer's protocol. Microglial cells were stimulated with LPS at 48 h and lysed at 72 h after transfection for Western blotting. For co-culture assay, 72 h after siRNA transfection, microglial cells were co-cultured with cortical neurons with LPS. The sense and antisense strands of RGMa siRNA were 5′-AACAGAUGCAGCUUGUCCUUGUUGG-3′ and 5′-CCAACAAGGACAAGCUGCAUCUGUU-3′, respectively.

### Nucleofection of mouse cortical neurons

Cortical neurons were suspended in 100 µl of Nucleofector solution (Amaxa Biosystems, Cologne, Germany) containing 250 pmol of mouse neogenin siRNA (Sigma-Aldrich) or negative control siRNA. Electroporation was performed by using program O-005 as described by the manufacturer's protocol (Amaxa Biosystems). Cortical neurons were lysed at 72 h after transfection for Western blotting. For co-culture assay, 72 h after siRNA transfection, cortical neurons were co-cultured with microglial cells. The sense and antisense strands of neogenin siRNA were 5′-CAAUUCCAUGGAUAGCAAUTT-3′ and 5′-AUUGCUAUCCAUGGAAUUGTT-3′, respectively.

### Western blot

The cells were lysed in lysis buffer containing 50 mM Tris–HCl (pH 7.8) with 150 mM NaCl, 1 mM EDTA, 2 mM Na_3_VO_4_, 1% NP-40, and protease inhibitor cocktail (Roche, Palo Alto, CA). The severed spinal cord (from 1 mm rostral to 1 mm caudal [2 mm long] to the lesion epicenter) was dissected and homogenized in lysis buffer. The lysates were incubated on a rocking platform at 4°C for 20 min and clarified by centrifugation at 13,000 *g* at 4°C for 20 min. Supernatants were collected, separated on SDS-PAGE gels, and transferred to a PVDF membrane (Millipore, Billerica, MA). The membranes were blocked with 5% nonfat dry milk in PBS containing 0.05% Tween-20 (PBST), then incubated with rat anti-RGMa antibody (1∶250; R&D, Minneapolis, MN), rabbit anti-neogenin antibody (1∶1000; Santa Cruz Biotechnology, Santa Cruz, CA), or mouse anti-actin antibody (1∶5000; Calbiochem, Darmstadt, Germany) overnight at 4°C. After being washed, membranes were incubated with HRP-linked anti-rat IgG antibody (1∶5000; Cell Signaling Technology, Beverly, MA), HRP-linked anti-rabbit IgG antibody (1∶5000; Cell Signaling Technology), or HRP-linked anti-mouse IgM antibody (1∶10000; Santa Cruz Biotechnology). Antibodies were detected using an ECL chemiluminescence system (GE Healthcare, Buckinghamshire, UK).

### Procedures for SCI and treatment

Eight-week-old adult female C57BL/6J mice were deeply anesthetized with somnopentyl (50 mg/kg, i.p.; Kyoritsu Seiyaku, Tokyo, Japan). A laminectomy was performed at T9 to expose the dorsal aspect of the spinal cord. Dorsal hemisection was performed at this level with a blade to completely sever the dorsal and dorsolateral corticospinal tracts (CSTs; depth: 1.0 mm). The muscle layers and skin on the back were then sutured. Minocycline (50 mg/kg, i.p.; Sigma-Aldrich) was administered for 7 or 14 consecutive days after the surgery. Control groups received equivolumetric intraperitoneal injections of PBS at the corresponding time points. All surgical procedures and postoperative care were performed in accordance with the guidelines of the Osaka University Animal Care and Use Committee.

### Anterograde labeling of the CST

One day or 1 week before the SCI, mice were anesthetized with somnopentyl (50 mg/kg, i.p.) and placed on a stereotaxic frame. Their scalps were incised so that the region of the skull overlying the sensorimotor cortex could be carefully split open with a drill. To label the CST, the anterograde tracer biotinylated dextran amine (BDA; MW, 10,000; 10% in PBS; Invitrogen), was injected at a total of 8 sites per individual (4 sites per side and 0.6 µl per site over 3 30-s injection cycles) at a depth of 0.5 mm from the cortical surface (coordinates: 1.0–1.5 mm posterior, 0.5–1.0 mm lateral to the bregma) by using a 5-µl microsyringe tipped with a pulled glass micropipette. After the injections, the skin overlying the skull was sutured.

### Immunohistochemistry

The animals received a transcardial perfusion of 4% PFA at 7 or 14 days after the SCI. Spinal cords were dissected and postfixed in the same fixative overnight. On the following day, the tissue was cryopreserved in 30% sucrose/PBS. Thoracic spinal cords were embedded in Tissue-Tek OCT compound (Sakura Finetek, Tokyo, Japan). Serial 14- or 20-µm-thick sagittal sections were cut with a cryostat and mounted on MAS-coated slides (Matsunami Glass, Osaka, Japan). Sections were blocked with 5% BSA/0.1% Triton X-100/PBS for 1 h and then incubated with the following primary antibodies overnight at 4°C: rat anti-CD11b (1∶250; Serotec, Kidlington, UK), rabbit anti-RGMa (1∶100; IBL), and mouse anti-phospho-neurofilament (SMI31; 1∶1000; Covance). Sections were washed with PBST, then incubated with the following secondary antibodies for 1 h at room temperature: Alexa Fluor 488 or Alexa Fluor 568 goat anti-rat IgG, goat anti-rabbit IgG, and goat anti-mouse IgG (1∶500; Invitrogen). For BDA labeling, the sections were incubated at room temperature in 5% BSA/0.1% Triton X-100/PBS for 1 h, then with Alexa Fluor 488-conjugated streptavidin (1∶500; Invitrogen) for 2 h. The sections were then washed with PBST and counterstained with DAPI (1 µg/ml; Santa Cruz Biotechnology) to allow visualization of the nuclei. All images were acquired with a fluorescence microscope (Olympus BX51, DP71; Tokyo, Japan) or a confocal laser-scanning microscope (Olympus FluoView FV1000).

Accumulation of microglia was assessed in three parasagittal CD11b-immunostained sections per animal. The positive area was measured by using ImageJ software and averaged. To quantify the dieback of the dorsal CST, the distance between the margin of lesion defined by accumulation of CD11b^+^ cells and the tips of the BDA-labeled CST axons (bulbous in appearance) was measured in one parasagittal section per animal (10 axons per section) and averaged. The border of accumulation of CD11b^+^ cells [22], which was determined automatically with ImageJ software (NIH), is approximately corresponded to the margin of lesion as the border between GFAP positive and negative site (astroglial and fibrotic scar), the definition used in previous studies [23], [24].

### Statistics

Quantitative data are expressed as the mean ± SEM. We investigated differences across all groups using one-way analysis of variance (ANOVA) tests, followed by Tukey-Kramer tests. We explored differences between pairs of experimental groups using Student's *t-*tests. Significance was defined as *P*<0.05.

## Results

### Activated microglia inhibit neurite outgrowth and induce growth cone collapse

**Figure 1:**
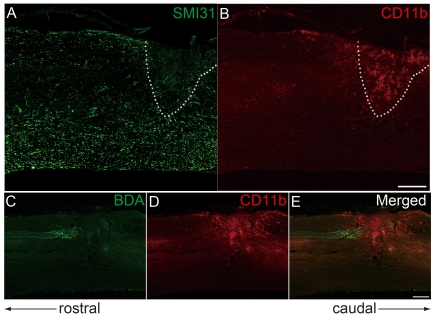
Transected axons do not regrow into the lesion of SCI where microglia/macrophages accumulate. *A*, *B*, Immunostaining of phospho-neurofilament (SMI31; *A*; axon, green) and CD11b (*B*; microglia/macrophage, red) at day 7 after SCI. Scale bar: 500 µm. *C*–*E*, BDA-labeled CST fibers (*C*, green) and CD11b (*D*; microglia/macrophage, red) at day 7 after SCI. Scale bar: 500 µm.

**Figure 2:**
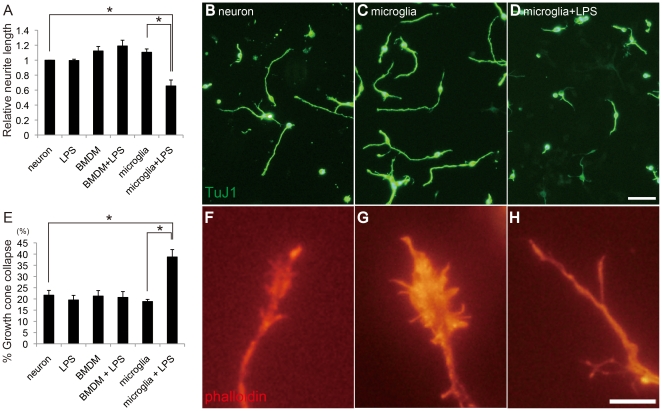
Activated microglia inhibit neurite outgrowth and induce growth cone collapse in vitro. *A*, Relative length of neurites of cortical neurons, neurons stimulated by LPS, and neurons co-cultured with BMDM, BMDM stimulated by LPS, microglia, or activated microglia stimulated by LPS (n = 3). *B*–*D*, Representative figures of neurons (TuJ1, green, *B*), neurons co-cultured with unactivated microglia (*C*), and neurons co-cultured with activated microglia (*D*). Scale bar: 20 µm. *E*, Collapse assay (n = 3). *F*–*H*, Representative figures of growth cones (phalloidin staining, red) of a neuron (*F*), a neuron co-cultured with unactivated microglia (*G*), and a neuron co-cultured with activated microglia (*H*). Scale bar: 10 µm. Data are represented as mean ± SEM. *p<0.01 (one-way ANOVA followed by Tukey-Kramer test).

At 7 days after SCI, transected axons were retracted and did not grow into the lesion site (Figure 1*A*). The lesion epicenter neighboring these retracted axons was composed of a number of CD11b^+^ cells, which were activated microglia or macrophages (Figure 1*A, B*). The transected CST, one of the major axonal tracts controlling fine motor function, was also retracted from the lesion site containing CD11b^+^ cells (Figure 1*C*–*E*). Thus, it appears likely that these cells affect nearby neurons. To assess the effect of microglia or macrophage on the neurons, we employed co-culture assay consisting of cortical neurons (E18) and microglia/macrophage *in vitro*. Co-culture assay revealed that neurite outgrowth tended to be slightly promoted when neurons were cultured with primary microglia or BMDM for 2 days, but statistically not significant (Figure 2*A*–*C*). However, when the neurons were cultured with microglia activated by LPS, neurite outgrowth was significantly inhibited (*P*<0.01; Figure 2*A*–*D*). Additionally, the growth cones of these neurons were collapsed (*P*<0.01; Figure 2*E*–*H*). LPS itself did not modulate neurite outgrowth and growth cone morphology (Figure 2*A, E*). On the other hand, BMDM activated by LPS did not inhibit neurite outgrowth, but rather slightly increased the neurite length than non-stimulated BMDM (statistically not significant; Fig. 2*A*). These results suggest that activated primary microglia, but not BMDM or unactivated microglia, inhibit neurite growth and induce growth cone collapse.

### Cell-cell contact is necessary for the inhibitory activity of activated microglia

**Figure 3:**
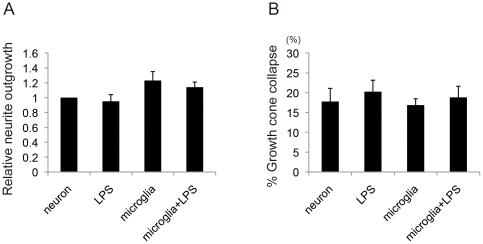
Cell-cell contact is necessary for activated microglia-induced neurite outgrowth inhibition and growth cone collapse. ***A***, Results of neurite outgrowth assay across 4 treatment groups (neurons-only, neurons + LPS, neurons co-cultured with unactivated microglia, neurons co-cultured with activated microglia) in the transwell system (n = 3). The neurons, seeded to the lower plane, were separately co-cultured with the unactivated and activated primary microglia, seeded to the upper plane. ***B***, Results of growth cone collapse assay across 4 treatment groups (neurons-only, neurons + LPS, neurons co-cultured with unactivated or activated microglia) in the transwell system (n = 3). Data are represented as mean ± SEM.

We hypothesized that some molecules expressed in the activated microglia inhibited neurite growth and collapsed growth cones. These molecules may be secreted from the activated microglia. To assess this, the neurons were separately co-cultured with the activated primary microglia in the transwell system. In contrast to our co-culture results (Figure 2), LPS-stimulated microglia had no effect on either neurite length or growth cone collapse (Figure 3*A, B*) in transwell plates. These results suggest that, adhesion molecules, rather than secreted molecules, should be responsible for the effects of microglia on neurite growth.

### RGMa mediates the effect of activated microglia

**Figure 4:**
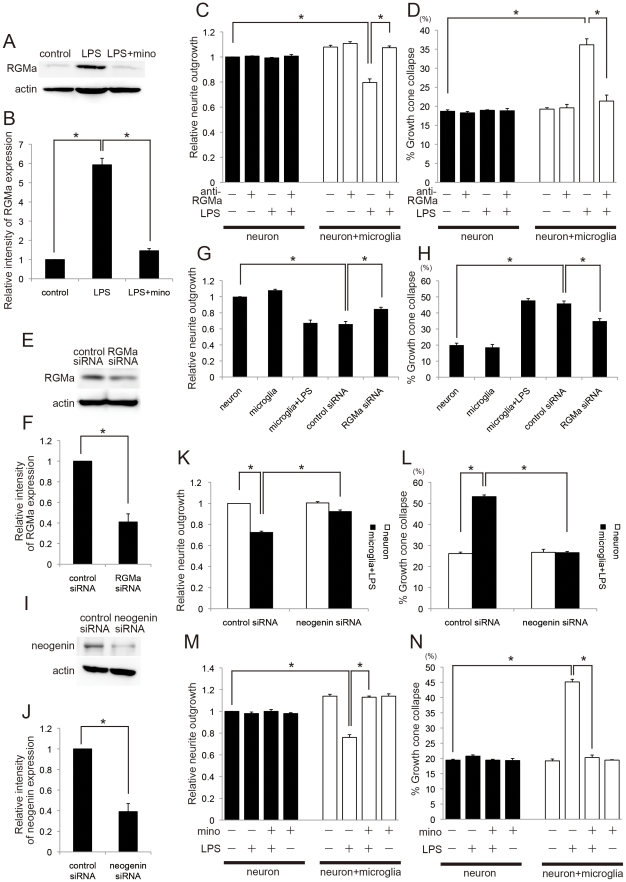
RGMa mediates the effect of activated microglia in inhibiting neurite outgrowth. *A*, Results of Western blot for RGMa in microglia. RGMa expression was higher in activated microglia (LPS) than in unactivated (control) or activated microglia treated with minocycline (LPS + mino). *B*, Quantitative data for *A* (n = 3). *C*, Effect of treatment with anti-RGMa antibody on the neurite outgrowth-inhibiting effects of activated microglia (n = 3). *D*, Effect of treatment with anti-RGMa antibody on activated microglia-induced growth cone collapse (n = 3). *E*, Results of Western blot for RGMa in RGMa siRNA- and control siRNA-transfected microglia stimulated by LPS. *F*, Quantitative data for *E* (n = 3). *G*, Relative length of neurites of cortical neurons and that co-cultured with microglia, activated microglia stimulated by LPS, activated microglia transfected with control siRNA (control siRNA), or activated microglia transfected with RGMa siRNA (RGMa siRNA) (n = 3). *H*, Collapse assay (n = 3). *I*, Results of Western blot for neogenin in neogenin siRNA- and control siRNA-transfected cortical neurons. *J*, Quantitative data for *I* (n = 3). *K*, Relative length of neurites of cortical neurons transfected with control siRNA or neogenin siRNA, and that co-cultured with activated microglia stimulated by LPS (n = 3). *L*, Collapse assay (n = 3). *M*, *N*, Effect of minocycline on activated microglia-induced neurite outgrowth inhibition (*M*, n = 3) and growth cone collapse (*N*, n = 3). Data are represented as mean ± SEM. *p<0.01 (*B*, *C*, *D*, *G*, *H*, *K*, *L*, *M*, *N*, one-way ANOVA followed by Tukey-Kramer test; *F*, *J*, Student's *t-*test).

Microglia express guidance molecules that are known to repel the axons, e.g. Slit, Netrin-1, and RGMa, in injured spinal cord [15], [16]. Given that microglia inhibit axonal growth via cell-cell contact (Figure 2, 3), we assessed if RGMa was involved in the effects. Indeed, RGMa was expressed in microglia and the expression was increased by LPS induction (*P*<0.01; Figure 4*A, B*). We employed anti-RGMa antibodies to attenuate the effect of RGMa [16], [25]. The addition of anti-RGMa antibodies dramatically reduced neurite outgrowth inhibition and growth cone collapse (*P*<0.01; Figure 4*C, D*). An antibody-only control treatment had no effect on neurite outgrowth or growth cone collapse. Next, we transfected RGMa siRNA or control non-targeting siRNA into primary microglia, and employed co-culture assay consisting of cortical neurons and microglia. RGMa siRNA efficiently reduced the expression of RGMa in activated microglia stimulated with LPS (*P*<0.01; Figure 4*E, F*). When the neurons were co-cultured with activated microglia transfected with RGMa siRNA, neurite outgrowth inhibition and growth cone collapse induced by activated microglia was decreased (*P*<0.01; Figure 4*G, H*). This effect was not observed in control siRNA-transfected microglia. These results indicate that RGMa mediates the effects of activated microglia on cortical neurons.

We further transfected neogenin, a receptor for RGMa, siRNA or control non-targeting siRNA into cortical neurons, and employed co-culture assay consisting of cortical neurons and microglia. Neogenin siRNA efficiently reduced the expression of neogenin in cortical neurons (*P*<0.01; Figure 4*I, J*). When the neurons transfected with neogenin siRNA were co-cultured with activated microglia, neurite outgrowth inhibition and growth cone collapse induced by activated microglia were decreased (*P*<0.01; Figure 4*K, L*). This effect was not observed in control siRNA-transfected neurons. These results indicate that RGMa-neogenin signal transduction is required for the effects of activated microglia on cortical neurons.

### Minocycline rescues activated microglia-induced neurite outgrowth inhibition and growth cone collapse

Minocycline inhibits microglial activation [12], [26]. This prompted us to investigate whether minocycline treatment could rescue the neurite growth inhibition induced by activated microglia and RGMa. Western blot analysis revealed that minocycline efficiently suppressed the increase of RGMa expression in activated microglia (*P*<0.01; Figure 4*A, B*). The neurons and microglia were co-cultured with or without the addition of minocycline (20 µM) for 2 days. It was found that neurite outgrowth inhibition and growth cone collapse were reduced to control levels in neuron-microglia co-cultures that were treated with minocycline (*P*<0.01; Figure 4*M, N*). Minocycline by itself had no effect on neurite outgrowth and growth cone collapse in the neurons. Therefore, minocycline inhibits the effects of activated microglia on the neurite outgrowth and RGMa expression.

### Blockage of microglial activation inhibits CST dieback with the reduction of RGMa expression

**Figure 5:**
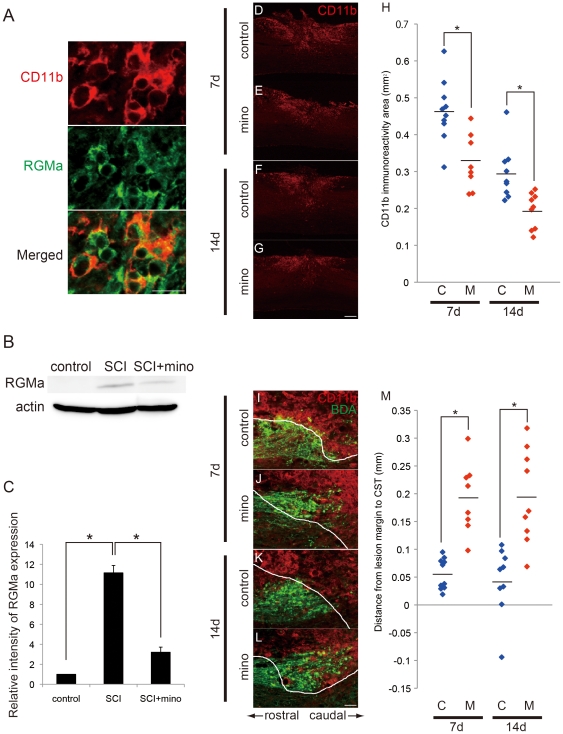
Blockage of micloglial activation by minocycline inhibits CST dieback with the reduction of RGMa expression after SCI. *A*, Expression of RGMa (green) by CD11b-positive microglia (red) in the lesion epicenter at 7 days after SCI. Scale bar: 20 µm. *B*, *C*, Changes in RGMa expression in response to SCI (day 7) and treatment with minocycline (n = 3). *p<0.01 (one-way ANOVA followed by Tukey-Kramer test). *D*–*G*, Changes in the number of CD11b^+^ cells (red) in an SCI lesion site at 7 (*E*) and 14 (G) days after treatment with minocycline. These patterns differ from those observed after treatment with the control vehicle only (*D*, *F*). Scale bar: 500 µm. *H*, Results of quantitative analyses investigating CD11b-positive area in minocycline-treated group (M) and vehicle-treated control (C) (7d, C, n = 10; M, n = 8; 14d, C, n = 9; M, n = 9). *p<0.05 (Student's *t-*test). *I*–*L*, Comparison of the effects of minocycline administration (*J*, *L*) and control vehicle treatment (*I*, *K*) on CST dieback at 7 and 14 days, respectively. CST axons (BDA, green) and the accumulating microglial cells (CD11b, red). White line indicates the margin of lesion (see Materials and methods). Scale bar: 100 µm. *M*, Quantitative analyses of CST dieback by minocycline treatment (M) and vehicle-treated control (C) (7d, C, n = 10; M, n = 8; 14d, C, n = 9; M, n = 9). Data are represented as mean ± SEM. *p<0.05 (Student's *t-*test).

We attempted to investigate the role of RGMa in microglia in vivo by employing mice SCI model. Intensive RGMa expression was found in CD11b-positive cells in the lesion epicenters of SCI mice; furthermore, this expression was significantly increased during the subacute phase of SCI (Figure 5*A*–*C*). Then, we administrated minocycline (50 mg/kg, i.p.) to mice for 7 or 14 consecutive days after SCI to inhibit microglial activation. Mice treated with minocycline accumulated significantly fewer CD11b-positive cells in their lesion sites (*P*<0.05; Figure 5*D*–*H*). Consistent with our observations *in vitro* (Figure 4*A, B*), minocycline treatment dramatically decreased the expression of RGMa, as measured at 7 days after SCI (*P*<0.01; Figure 5*B, C*).

Then, we assessed the morphological changes in the transected CST. We observed dieback of CST from the lesion site at 7 and 14 days after SCI; however, this distance was significantly decreased by the minocycline treatment (*P*<0.05; Figure 5*I*–*M*). These results support our hypothesis that RGMa mediates the effects of activated microglia on axonal dieback.

## Discussion

Our findings provide evidence that microglia are one of the major factors inhibiting axonal regeneration in the CNS after SCI. Furthermore, we have shown that this function of microglia is mediated by RGMa. To our knowledge, this is the first report identifying the molecule that is responsible for microglia-mediated axonal growth inhibition.

Ours are not the only results highlighting the detrimental effects of microglia after SCI [6], [10]; however, a number of studies have shown that microglia/macrophages can also have a positive effect during this period [4], [5]. The conflict between these 2 sets of findings may stem from grouping together subsets of microglia/macrophages that have distinct roles during neuronal regeneration. Specifically, macrophages can be classified into at least 2 categories: “classically activated,” or M1, and “alternatively activated,” or M2 [27], [28]. The M1 phenotype produces a high quantity of pro-inflammatory cytokines and free radicals that are essential for host defense, while the M2 phenotype repairs tissues and reduces inflammation by regulating the inflammatory response. This classification scheme can also be applied to microglia [29].

Both M1 and M2 macrophages accumulate in the injured spinal cord, but while M1 macrophages are neurotoxic, M2 macrophages are neuroprotective [9]. Thus, the M1/M2 ratio may determine whether CNS macrophages are ultimately detrimental or helpful to axonal regeneration after SCI. Indeed, it has been reported that infiltrating monocyte-derived macrophages expressing interleukin 10 (M2) aid recovery from SCI [8]. Nevertheless, M1 becomes increasingly more common as time elapses after SCI, ultimately reducing neuronal survival and axonal growth [9].

In the current study, RGMa expression in microglia, and therefore inhibition of neurite growth, was increased after the primary microglia were stimulated by LPS, which promotes differentiation into the M1 phenotype. Thus, our results highlight one of the important effects of M1 phenotype and its molecular mechanism detrimental to the lesion, i.e. RGMa-mediated inhibition of axonal regeneration. In addition, our data show that activated microglia, but not macrophages, inhibit axonal growth. This partly contradicts the findings of Horn et al. (2008) [10], who reported that activated macrophages, as well as activated microglia, induce retraction of the dystrophic axons of dorsal root ganglionic neurons. The discrepancy between these findings may be a result of the fact that, whereas Horn et al. (2008) examined dystrophic axons, our experiments focused on healthy cortical neurons.

The results of our transwell culture assay suggest that cortical neurons are not affected by factors secreted from microglia. This observation is consistent with previous reports [10]; further, our hypothesis that microglia-derived RGMa inhibits axonal regeneration in the CNS is supported by several previous findings. First, RGMa inhibits axonal outgrowth and facilitates growth cone collapse [16], [17], [21]. Second, RGMa levels increase after SCI, as a result of increased expression by activated microglia/macrophages (Figure 5*A*–*C*; [16]). Third, administration of anti-RGMa antibody promotes axonal growth and functional recovery after SCI [16]. Fourth, minocycline treatment decreases the number of microglia/macrophages and expression of RGMa, leading to suppression of axonal retraction (dieback). In order to definitely demonstrate the importance of RGMa in the process of axon regrowth inhibition, future studies will need to investigate the impacts of selectively deleting this gene from microglia.

We used minocycline to block the activation of microglia. Treatment with minocycline improves functional outcome after SCI [12], [30]–[32]. The beneficial effects of minocycline are at least partly dependent on suppression of cell death; for example, minocycline prevents oligodendrocyte death by inhibiting proNGF production in microglia [32], and is postulated to inhibit neuronal cell death by attenuating TNF-α and NO production [33]. Like Stirling et al. (2004) [12], we found that minocycline treatment suppresses microglia/macrophage accumulation in SCI lesions and reduces axonal dieback of the CST in a mouse model. More importantly, we have also shown that minocycline rescues the inhibitory role of activated microglia on axonal growth in vitro, and that this effect is mediated through reduction of RGMa expression.

To investigate whether minocycline treatment has noticeable effects on mouse behavior, we evaluated the recovery of hindlimb locomotor function using Basso mouse scale (BMS) scores for 2 weeks post-SCI. We did not detect any significant differences in improvement between control and treated mice (data not shown), a finding that is consistent with some reports [34], [35], but inconsistent with others [12], [30]–[32]. This discrepancy may be due to differences in experimental conditions, divergent functions of microglia, and the effects of minocycline on other cells.

In our experimental paradigm, minocycline treatment suppressed the retraction of CST but could not induce robust axonal regeneration beyond the lesion or sprouting (Figure 5). Because many inhibitory mechanisms are thought to collectively affect the capacity for regeneration in adult CNS, a combinatory approach that allows the elimination of a large number of inhibitory cues [1], [14] and the promotion of neurons' intrinsic axon-growing capacity [3] will be required to reconstruct the injured neural network. Our study makes an important first step in this process by elucidating the function of activated microglia and describing the mechanism underlying their activity.
